# 1572. Impact of a Multicenter Fluoroquinolone Stewardship Collaboration on Prescribing Trends over Five Years

**DOI:** 10.1093/ofid/ofac492.101

**Published:** 2022-12-15

**Authors:** Kelsey Olson, Laura Blackburn, Judy Feltman

**Affiliations:** HCA Houston Healthcare Clear Lake, Houston, TX; HCA Houston Healthcare Clear Lake, Houston, TX; HCA Houston Healthcare, Houston, Texas

## Abstract

**Background:**

Fluoroquinolone (FQ) use is generally discouraged in uncomplicated infections when alternative therapies exist. Antimicrobial stewardship programs use diverse strategies to encourage appropriate prescribing of antibiotics, and FQ stewardship is often a priority due to the known toxicity risks. This study aimed to evaluate how the coordinated implementation of several stewardship strategies impacted FQ prescribing at eight community hospitals.

**Methods:**

This retrospective, observational study includes data from January 2017 to January 2022. In 2018, the facility clinical pharmacy programs established formal clinical metrics to be tracked and reported on a regular basis. One such metric tracked FQ prescribing in urinary tract infections (UTI), and this metric has remained active since 2018. In early 2019, the health-system implemented a clinical decision support alert in the electronic health record, which requires acknowledgement of a risk/benefit assessment upon FQ order entry. In late 2019, some facilities began suppressing automated susceptibility results for FQ on cultures, due to technological limitations with new minimum inhibitory concentration breakpoints. Using a clinical surveillance system, a report was generated to assess the year-over-year changes in FQ days of therapy (DOT) per 1000 patient days at each facility.

**Results:**

An 83% reduction in FQ use was observed over five years (737 DOT/1000 patient days vs. 125 DOT/1000 patient days), with facility-level reductions ranging from 62% to 96%. The smallest decrease in FQ use (18%) occurred from 2017–2018, before any system-wide FQ stewardship initiatives were adopted. A larger decrease in FQ use (33%) was seen from 2018–2019, after introducing the formal FQ UTI metric. The biggest decrease in FQ use (50%) occurred from 2019–2020, when the FQ alert and susceptibility suppression strategies were implemented alongside the existing UTI metric. An additional 39% decrease in FQ use was seen from 2020–2022, when no additional FQ interventions were implemented.

**Conclusion:**

Substantial decreases in FQ prescribing were observed when multiple FQ stewardship strategies were utilized. The decreases were sustained over several years across multiple hospitals.

**Disclosures:**

**All Authors**: No reported disclosures.

